# A Neutral Polysaccharide from Spores of *Ophiocordyceps gracilis* Regulates Oxidative Stress via NRF2/FNIP1 Pathway

**DOI:** 10.3390/ijms241914721

**Published:** 2023-09-29

**Authors:** Yue Wang, Shixiang Wei, Hui Lian, Lingling Tong, Linhui Yang, Bo Ren, Dongsheng Guo, He Huang

**Affiliations:** School of Food Science and Pharmaceutical Engineering, Nanjing Normal University, NO 1, Wen Yuan Road, Nanjing 210023, China

**Keywords:** *Ophiocordyceps gracilis*, spores, polysaccharide, structural characterization, antioxidant activity

## Abstract

*Ophiocordyceps gracilis* (*O. gracilis*) is a parasitic fungus used in traditional Chinese medicine and functional foods. In this study, a neutral heteropolysaccharide (GSP-1a) was isolated from spores of *O. gracilis*, and its structure and antioxidant capacities were investigated. GSP-1a was found to have a molecular weight of 72.8 kDa and primarily consisted of mannose (42.28%), galactose (35.7%), and glucose (22.02%). The backbone of GSP-1a was composed of various sugar residues, including →6)-α-D-Manp-(1→, →2,6)-α-D-Manp-(1→, →2,4,6)-α-D-Manp-(1→, →6)-α-D-Glcp-(1→, and →3,6)-α-D-Glcp-(1→, with some branches consisting of →6)-α-D-Manp-(1→ and α-D-Gal-(1→. In vitro, antioxidant activity assays demonstrated that GSP-1a exhibited scavenging effects on hydroxyl radical (^•^OH), 2,2′-azino-bis-3-ethylbenzothiazoline-6-sulfonic acid radical cation (ABTS^•+^), and 2,2-diphenyl-1-picrylhydrazyl radical (DPPH^•^). Moreover, GSP-1a was found to alleviate H_2_O_2_-induced oxidative stress in HepG2 cells by reducing the levels of reactive oxygen species (ROS) and malondialdehyde (MDA), while enhancing the activities of superoxide dismutase (SOD). Furthermore, GSP-1a upregulated the mRNA expression of antioxidant enzymes such as *Ho-1*, *Gclm*, and *Nqo1*, and regulated the NRF2/KEAP1 and FNIP1/FEM1B pathways. The findings elucidated the structural types of GSP-1a and provided a reliable theoretical basis for its usage as a natural antioxidant in functional foods or medicine.

## 1. Introduction

*Cordyceps* belong to Ascomycotina and is a parasitic fungus that grows on the larva or pupae of insects [1]. *Cordyceps* have a broad range of various pharmacological properties, where *Ophiocordyceps sinensis* (*O. sinensis*) is the most well-known and has been extensively used in Asian countries as a tonic and medicinal food [2]. In addition, *Ophiocordyceps gracilis* (*O. gracilis*) parasitizes the larvae of *Hepialus*, which belongs to the same genus and has a similar chemical composition and medicinal properties with *O. sinensis* and has been listed in the drug standards of Xinjiang China [3,4]. These might indicate that *O. gracilis* could have interesting medicinal properties, some of which also have been recently described [5,6]. Furthermore, *Cordyceps* has significant therapeutic effects on a variety of diseases, including renal, hepatic, respiratory, neurological, and cardiovascular diseases, cancer, aging, and hyperlipidemia [7,8,9,10]. The pharmacological components of *Cordyceps* include cordycepin, polysaccharides, bioactive peptides, mannitol, and ergosterol [2]. Among these constituents, polysaccharides are the major active compounds in *Cordyceps* and exhibit a broad range of biological activities [11,12,13,14]. Notably, the antioxidant property is one of the significant activities of Cordyceps polysaccharides, which would be also one of the mechanisms for its physiological function. Oxidative stress is associated with various diseases, such as cancer, cardiovascular disease, type-II diabetes, immunity diseases, and aging [15,16]. Reactive oxygen species (ROS) scavenging improves oxidative homeostasis and delays the onset and progression of aging and other diseases [12]. At present, many synthetic antioxidants are widely used, but they are unable to consistently prevent ROS-induced damage in vivo and can even increase the incidence of diseases [17]. Therefore, there is an urgent need to develop safe and effective natural antioxidants as alternatives to synthetic ones. *Cordyceps* polysaccharides possess excellent biocompatibility and non-toxic characteristics, making them easily tolerated by the human body. Consequently, the application of *Cordyceps* polysaccharides is a rising concern in the defense against a variety of oxidative stresses.

Previously, studies have tended to focus on natural *Cordyceps* polysaccharides as well as intracellular and extracellular polysaccharides of cultured *Cordyceps* rather than those originating from spores of *Cordyceps* [18,19]. Fungal spores contain all the genetic substances, possess similar bioactive components to the fruiting body, and even have greater pharmaceutical values than the fruiting body, such as *Ganoderma lucidum* spores. *Ganoderma lucidum* spore polysaccharides have been reported to contain various physiological and health effects, such as strong antioxidant activities, immunomodulating activities, and anti-tumor activities [20,21]. It is worth noting that the State Drugs Administration of China has approved the use of *Ganoderma lucidum* spore polysaccharides injection (GuoYaoZhunZi H20003510 and H20003123) for the treatment of neurosis, progressive muscular dystrophy, and various diseases caused by a compromised immune system [22]. These suggest that polysaccharides from spores of medicinal mushrooms are important resources for functional food development and new drug discovery. Studies have shown that polysaccharides from spores of *Cordyceps cicadae* exhibited a higher ability to promote glucose absorption, reduce insulin resistance, and improve type II diabetes compared to mycelia and sclerotia polysaccharides [23]. In addition, polysaccharides from spores of *Cordyceps cicadae* exhibited ameliorative effects in immunosuppressed mice through enhancing macrophage phagocytic activity, improving natural killer cytotoxicity, and modulating antioxidant enzyme system [22]. However, polysaccharides from *Cordyceps* spores are less studied. In our previous studies, large quantities of *O. gracilis* were obtained in submerged culture via the microcycle conidiation technique [24]. Numerous studies have shown that the functions of polysaccharides are closely linked to their structural characteristics, such as monosaccharide composition, molecular weight (Mw), and chemical linkages [25,26]. Therefore, it is crucial to understand the structure of polysaccharides originating from the spores of *O. gracilis*.

In this study, a neutral polysaccharide (GSP-1a) was isolated and purified from *O. gracilis* spores. Its structural characteristics, including molecular weight, monosaccharide composition, and glycosyl linkages, were elucidated, and the plausible structure was predicted. In addition, the extracellular antioxidant activity of GSP-1a was evaluated by free radical scavenging assays (^•^OH, ABTS^•+^, DPPH^•^). Furthermore, the protective effects of GSP-1a on H_2_O_2_-induced oxidative stress in HepG2 cells were investigated via cellular antioxidant activity assays and its possible mechanism was revealed. This work aims to help understand the structural characteristics of *O. gracilis* spore polysaccharides and provide a theoretical reference for the development of new antioxidants and functional foods or medicine.

## 2. Results and Discussion

### 2.1. Molecular Weight and Chemical Composition of GSP-1a

**Figure 1:**
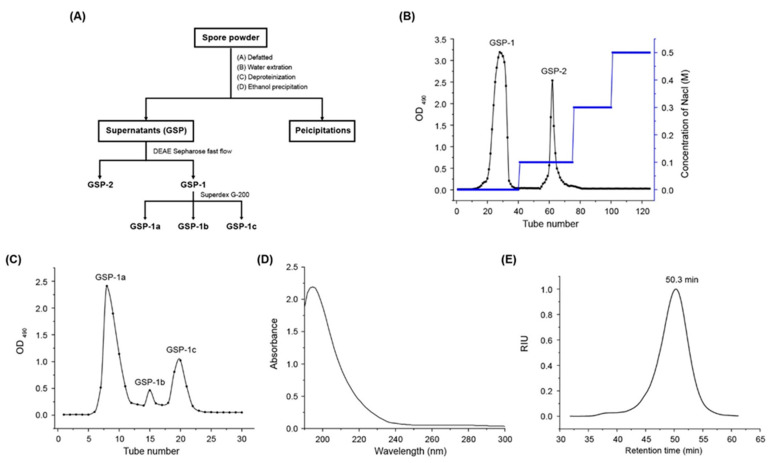
The procedure of GSP-1a extraction and purification. (**A**) Elution profile of GSP on DEAE Sepharose fast flow column. (**B**) Elution profile of GSP-1 on Superdex G-200 column. (**C**) UV–Vis spectrum of GSP-1a. (**D**) HPGPC profile of GSP-1a (**E**).

The flow diagram for the isolation and purification of polysaccharides from *O. gracilis* spores is shown in Figure 1A. The extraction of crude polysaccharides (GSP) yielded 7.9%. Then GSP was dissolved in ultrapure water and loaded onto a DEAE Sepharose fast flow column (2.6 cm × 40 cm) with gradient elution of 0–0.3 M NaCl solutions. Figure 1B illustrates that two fractions GSP-1 and GSP-2 were eluted under 0 and 0.1 mol/L NaCl solution with a yield of 55% and 10%, respectively. The first peak, GSP-1, represented the major fraction of GSP, which was further purified using the Superdex G-200 column. Figure 1C shows the composition of GSP-1, primarily consisting of GSP-1a (61%), GSP-1b (3%), and GSP-1c (16%). This indicated that GSP-1a was the dominant fraction, thus warranting further analysis of its structure. UV–Vis spectra (Figure 1D) revealed the absence of absorption peaks at 260 nm and 280 nm, indicating the absence of protein and nucleic acids in GSP-1a. Furthermore, gel permeation chromatography (Figure 1E) demonstrated a single and symmetrical peak for GSP-1a, indicating its homogeneity. The molecular weight (Mw) of GSP-1a was determined to be 72.8 kDa, with an Mw/Mn of 1.1 (Table 1), indicating that GSP-1a was a homogeneous polysaccharide.

The HPAEC quantitative analysis of the monosaccharide composition of GSP-1a is shown in Table 1 and Figure S1. Comparing the chromatogram of the mixed standard monosaccharides with that of monosaccharide components in GSP-1a, it could be seen that GSP-1a mainly consisted of mannose (man, 42.28%), galactose (gal, 35.7%), and glucose (glc, 22.02%). The monosaccharide components of GSP-1a were different from those of *Ganoderma lucidum* spore polysaccharides (glucose:galactose:arabinose = 90.82:7.95:1.23, molar percentage), *Paecilomyces cicadae* spore polysaccharides (glucose:galactose:mannose:arabinose = 8:5:4:1, molar ratio), and *Cordyceps cicadae* spores polysaccharides (CCSP-2 glucose:mannose = 94.27:5.73, CCSP-3 xylose:mannose:glucose:galactose = 22.08:2.05:63.4:12.27, molar percentage) [21,22,27]. This suggests that there are significant differences in the structure of polysaccharides from the spores of different mushroom species.

### 2.2. FT–IR Analysis of GSP-1a

**Figure 2:**
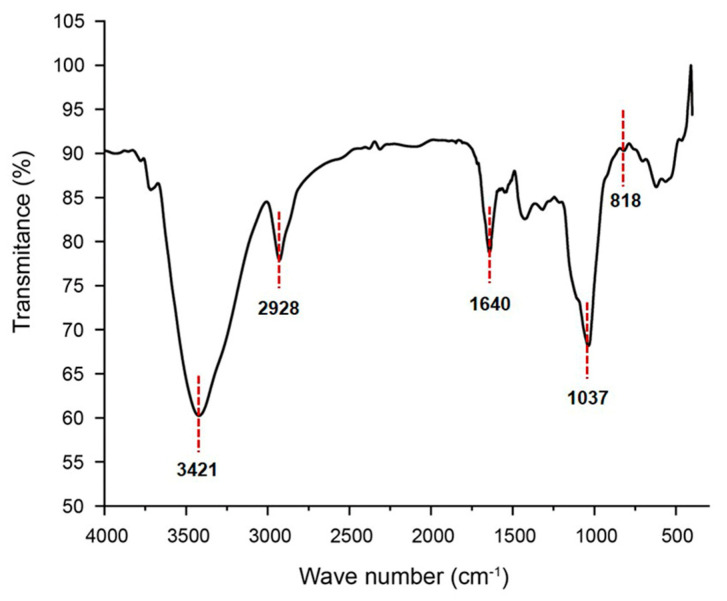
The FT–IR spectrum of GSP-1a.

Fourier transform infrared spectroscopy (FT–IR) spectrum of GSP-1a, presented in Figure 2, exhibited several characteristic peaks. The strong and broad peak at approximately 3421 cm^−1^ was assigned to the O-H stretching vibration, a typical feature of carbohydrates [28]. The signal at 2928 cm^−1^ corresponded to the stretching vibration of the C-H bonds in the sugar ring [29]. Additionally, the absorption between 1400 and 1200 cm^−1^ was indicative of carbohydrate-specific features [30]. The absence of a peak around 1730 cm^−1^ indicated the absence of uronic acid [13], which was consistent with the monosaccharide composition analysis. The peak at 1642 cm^−1^ may correspond to the hydroxyl group in the bound water [31]. The strong peak detected at approximately 1037 cm^−1^ represented the bending vibration of C-OH [32]. The signals in the range of 1000–1200 cm^−1^ were attributed to the stretching vibrations of C-O-C and C-O-H linkages [33]. Furthermore, the peak at 820 cm^−1^ indicated the presence of α-glycosidic bonds [34], which was consistent with the results of nuclear magnetic resonance (NMR) spectroscopy.

### 2.3. Methylation Analysis of GSP-1a

To ensure the linkage patterns of monosaccharides, GSP-1a was completely methylated; thereafter, the retention time, peak values, and ion fragment characteristics of partially methylated alditol acetates (PMAAs) products were detected using GC-MS and compared with a database [26]. The linkage analysis of GSP-1a is summarized in Table 2. For the polysaccharide, the non-reducing terminals consisted of Galp (34.36%). The branching points were at 3, 6-Glcp (9.6%), 2, 6-Manp (15.46%), and 2, 4, 6-Manp (5.09%), indicating that GSP-1a had a certain branching structure. Other residues were disubstituted, including 6-Manp (19.99%), 6-Glcp (9.87%), a small amount of 2-Manp (1.95%), and 4-Glcp (3.68%). Overall, the results of methylation were consistent with the findings of monosaccharide composition analysis.

### 2.4. NMR Spectroscopy Analysis of GSP-1a

To further interpret the structure of GSP-1a, the polysaccharide was analyzed via 1D-NMR (^1^H NMR and ^13^C NMR) and 2D-NMR (^1^H−^1^H COSY, ^1^H−^13^C HSQC, ^1^H−^1^H NOESY, and ^1^H−^13^C HMBC). NMR spectroscopy is a powerful tool for obtaining detailed structural information for carbohydrates. It can aid identifying the monosaccharide composition, determining the α- or β-anomeric configurations, and establishing linkage patterns and sequences of sugar units in polysaccharides [35]. H/C chemical shifts in all sugar residues were fully assigned based on the NMR spectra data and literature.

**Figure 3:**
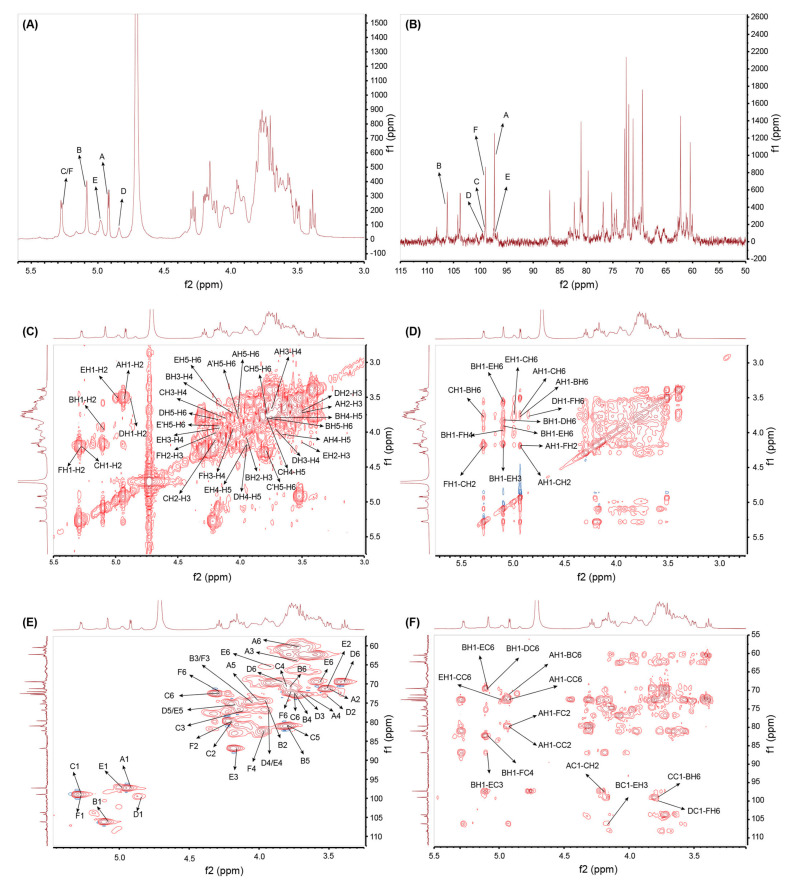
^1^H NMR (**A**), ^13^C NMR (**B**), ^1^H−^1^H COSY (**C**), ^1^H−^1^H NOSEY (**D**), ^1^H−^13^C HSQC (**E**), and ^1^H−^13^C HMBC (**F**) spectrum of GSP-1a.

The signals for GSP-1a in ^1^H and ^13^C NMR spectra were presented in Figure 3A,B. The proton signals were observed in the region ranging from δ 3.0 to 5.5 ppm, while the carbon signals appeared in the range of δ 90 to 110 ppm, which were the typical chemical shifts of polysaccharides [36]. In the ^1^H NMR spectrum, a strong signal at δ 4.71 ppm was assigned to the solvent proton peak, serving as a reference for other peaks. Six anomeric proton signals were observed at δ 4.92 ppm, δ 5.08 ppm, δ 5.27 ppm, δ 4.84 ppm, δ 4.98 ppm, and δ 5.27 ppm, corresponding to the H-1 positions of residues A–F, respectively. Furthermore, the corresponding anomeric carbon signals were observed at δ 97.29 ppm, δ 106.28 ppm, δ 98.97 ppm, δ 99.56 ppm, δ 97.24 ppm, and δ 99.08 ppm, as determined from the ^13^C NMR spectrum. According to the methylation analysis results, two sugar residues were not shown in NMR, which may be due to their low content. Additionally, the absence of signals in the range of δ 160–180 ppm in the ^13^C NMR spectrum indicates that GSP-1a does not contain uronic acid, confirming its classification as a neutral polysaccharide [37]. The ^1^H−^1^H COSY spectrum was employed to analyze the interconnectivity between adjacent hydrogen atoms within the same carbon atom [38]. On the other hand, the ^1^H−^13^C HSQC spectrum was utilized to study the distribution of ^1^H and ^13^C coupling within the same glycosidic bond [39]. By utilizing these spectra, the chemical shifts of glycosidic bonds A, B, C, D, E, and F were fully assigned based on the correlation peaks observed in the ^1^H−^1^H COSY and ^1^H−^13^C HSQC spectra.

For residue A, the cross-peaks were found at δ 4.92/3.50, δ 3.50/3.74, δ 3.74/3.66, δ 3.66/4.01, and δ 4.01/3.71, 3.80 in the ^1^H−^1^H COSY spectrum (Figure 3C), indicating that H2−H6 signals of residue A were at δ 3.50 ppm, δ 3.74 ppm, δ 3.66 ppm, δ 4.01 ppm, and δ 3.71/3.80 ppm. Similarly, the cross-peaks were at δ 3.50/71.24, δ 3.74/72.09, δ 3.66/72.68, δ 4.01/74.37, and δ 3.71/3.80, 60.39 in ^1^H−^13^C HSQC (Figure 3E), showing that C2−C6 resonances were at δ 71.24 ppm, δ 72.09 ppm, δ 72.68 ppm, δ 74.37 ppm, and δ 60.39 ppm. Combined with methylation results and references, it was speculated that the glycosidic bond A might be α-D-Galp-(1→[40]. Furthermore, the H2−H6 signals and C2−C6 signals of six anomeric residues of GSP-1a were obtained according to the ^1^H−^1^H COSY and ^1^H−^13^C HSQC spectra. All the hydrogen and carbon signals are listed in Table 3. The H-2/C-2 to H-6/C-6 of residue B were δ 3.95/74.37, 4.05/74.72, 3.75/72.34, 3.78/80.94, and 3.77/70.65 ppm, respectively. The H-2/C-2 to H-6/C-6 of residue C were δ 4.19/79.72, 4.11/76.88, 3.79/70.65, 3.78/80.94, and 3.75/72.36 ppm, respectively. The H-2/C-2 to H-5/C-5 of residue D were δ 3.50/73.06, 3.71/72.81, 3.94/76.75, 4.17/75.31, and 3.39/69.46 ppm, respectively. The H-2/C-2 to H-5/C-5 of residue E were δ 3.51/71.03, 4.16/86.89, 3.94/76.75, 4.17/75.31, and 3.91/69.59 ppm, respectively. The H-2/C-2 to H-4/C-4 of residue F were δ 4.19/79.72, 4.05/74.72, and 3.96/82.23, respectively.

Residue B exhibited a strong signal of anomeric proton and an anomeric carbon signal in the ^1^H NMR, ^13^C NMR, and ^1^H−^13^C HSQC spectra, suggesting its high content and proportion within GSP-1a. Based on the methylation analysis and monosaccharide component results, residue B was inferred to be an α-linked mannose (Man) residue. Further, according to the corresponding references, we speculated that the glycosidic bond B might be →6)-α-D-Manp-(1→[26,41,42,43]. In addition, combined with the methylation results and the relevant literature, it was inferred that the glycosidic bonds C, D, E, and F were→2,6)-α-D-Manp-(1→, →6)-α-D-Glcp-(1→, →3,6)-α-D-Glcp-(1→ and →2,4,6)-α-D-Manp-(1→[37,41,44,45,46,47].

**Figure 4:**
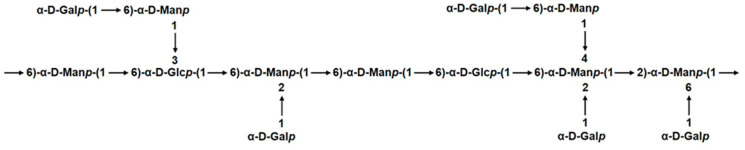
Predicted model structure of GSP-1a.

The glycosidic linkage sequence among the sugar residues of GSP-1a was determined through correlation peaks obtained in the ^1^H−^13^C HMBC and ^1^H−^1^H NOESY spectrum. The ^1^H-^13^C HMBC spectrum shows carbon–hydrogen coupling between different sugar residues [38]. As shown in Figure 3F, a series of inter-residual correlations were found among residues. The cross peaks between H-1 of residue A and C-6 of residue B, H-1 of residue A and C-2 of residue C/F, and C-1 of residue A and H-2 of residue C/F indicated the presence of sequence of α-D-Galp-(1→6)-α-D-Manp-(1→, α-D-Galp-(1→2,6)-α-D-Manp-(1→ and α-D-Galp-(1→2,4,6)-α-D-Manp-(1→. The cross peaks between H-1 of residue B and C-6 of residue D/E, H-1 of residue B and C-3 of residue E, H-1 of residue B and C-4 of residue F, and C-1 of residue B and H-3 of residue E indicated the presence of sequence of →6)-α-D-Manp-(1→6)-α-D-Glcp-(1→, →6)-α-D-Manp-(1→3,6)-α-D-Glcp-(1→ and →6)-α-D-Manp-(1→6,3)-α-D-Glcp-(1→. The cross peak of C-1 of residue C and H-6 of residue B indicated the presence of sequence of →2,6)-α-D-Manp-(1→6)-α-D-Manp-(1. The cross peak of C-1 of residue D and H-6 of residue F indicated the presence of sequence of →6)-α-D-Glcp-(1→6,2,4)-α-D-Manp-(1→. The cross peak of H-1 of residue E and C-6 of residue C indicated the presence of sequence of →3,6)-α-D-Glcp-(1→6,2)-α-D-Manp-(1→. All the above inter-residual correlations were also found via NOSEY. Moreover, the cross-peaks between H-1 of residue A and H-6 of residue C, as well as H-1 of residue F and H-2 of residue C, were identified (Figure 3D), indicating the presence of two sequence α-D-Galp-(1→6,2)-α-D-Manp-(1→ and →2,4,6)-α-D-Manp-(1→2,6)-α-D-Manp-(1→. Therefore, the backbone of GSP-1a appeared to be mainly composed of →6)-α-D-Manp-(1→, →2,6)-α-D-Manp-(1→, →2,4,6)-α-D-Manp-(1→, →6)-α-D-Glcp-(1→, and →3,6)-α-D-Glcp-(1→ residues with some branches consisting of →6)-α-D-Manp-(1→ and α-D-Gal-(1→ residues. A hypothetical model structure of GSP-1a was inferred according to the comprehensive analysis of FT–IR, monosaccharide composition, methylation, and NMR, as shown in Figure 4.

### 2.5. Antioxidant Activities In Vitro of GSP-1a

**Figure 5:**
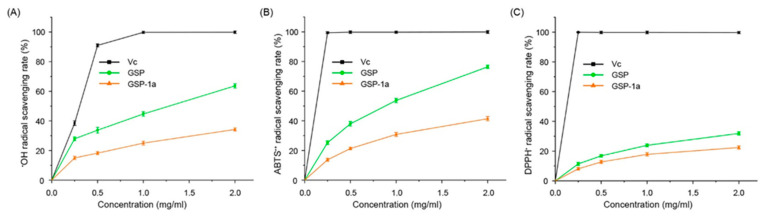
The ^•^OH (**A**), ABTS^•+^ (**B**), and DPPH^•^ (**C**) radical scavenging ability of GSP-1a and GSP. Vc is the positive control. The data are expressed as the mean ± SD (*n* = 3).

Excessive accumulation of free radicals in organisms can lead to a variety of diseases, particularly aging and age-related illnesses. Natural polysaccharides have gained widespread attention due to their excellent antioxidant activity, nontoxic, stable, and biocompatibility, especially fungal and plant polysaccharides [48]. Therefore, evaluating the antioxidant capacities of compounds of GSP-1a is crucial. In this study, we conducted ^•^OH, ABTS^•+^, and DPPH^•^ radical scavenging assays to evaluate the antioxidant capacities of GSP-1a, comparing them with the antioxidant activity of vitamin C (Vc). The ^•^OH is considered a strong reactive oxygen radical that can react with most biological molecules, causing tissue damage and cell death; so, removing hydroxyl radicals is vital to protect living systems [49]. As shown in Figure 5A, both GSP-1a and GSP exhibited a dose-dependent scavenging effect on ^•^OH radical. Within the concentration range of 0.25–2.0 mg/mL, the scavenging activity of GSP increased linearly from 27.9% to 63.6%. The half maximal inhibitory concentration (IC50) value of GSP was 1.25 mg/mL. While GSP-1a was less effective in scavenging ^•^OH radicals compared with GSP, the scavenging activity was 34.2% in 2 mg/mL. The ABTS^•+^ radical scavenging method is widely used to evaluate the total antioxidant ability of natural products [50]. As presented in Figure 5B, the trends for ABTS^•+^ radical scavenging activities of GSP-1a and GSP were similar. The scavenging activity of GSP-1a was enhanced from 13.7% to 41.4% with increasing concentration in the range of 0.25–2.0 mg/mL, while that of GSP was enhanced dramatically from 25.2 ± 1.2% to 76.4 ± 1.1%. The DPPH^•^ radical scavenging assay relies on the ability of antioxidants to donate hydrogen, and it is widely used as a means of estimating the free-radical scavenging activities of antioxidants [51]. Polysaccharides possess the unique capability to function as electron donors, allowing them to interact with free radicals and convert them into more stable forms [52]. This antioxidant activity enables polysaccharides to play a vital role in neutralizing and scavenging free radicals. As depicted in Figure 5C, the scavenging activity of both GSP-1a and GSP increased in a concentration-dependent manner. At a concentration of 2 mg/mL, GSP-1a exhibited a scavenging activity of 22.4%, while GSP demonstrated a scavenging activity of 32%.

Overall, the antioxidant activity of GSP-1a and GSP was lower than that of Vc at concentrations ranging from 0.25 to 2 mg/mL. However, the analysis of ^•^OH, ABTS^•+^, and DPPH^•^ radical scavenging activities still revealed that GSP-1a and GSP exhibit significant antioxidant activity in vitro. The antioxidant capacity of GSP-1a is comparable to that of certain *Cordyceps* polysaccharides with similar molecular weights and monosaccharide compositions. For example, when treated with a 2 mg/mL concentration, *O. sinensis* polysaccharides with a molecular weight of 28 kDa (backbone composed by α-1,4-Glc) and 43 kDa (man:gal:glc = 1.97:1:15.63, molar ratio) displayed ^•^OH scavenging capacities of 30% and 20%, respectively [53,54]. At a concentration of 1 mg/mL, the *Cordyceps cicadae* polysaccharides with a molecular weight of 60.7 kDa (gal:glu:man = 0.89:1:0.39, molar ratio) exhibited similar ^•^OH and ABTS^•+^ scavenging capacities of 20% and 30%, respectively [55]. Furthermore, the scavenging capacity for DPPH^•^ and ^•^OH of GSP-1a is superior to that of neutral polysaccharides with a molecular weight of 20 kDa from four types of mushrooms (*Pleurotus eryngii*, *Flammulina velutipes*, *Pleurotus ostreatus*, and white *Hypsizygus marmoreus*) [56]. It is worth noting that the crude polysaccharide fraction (GSP) tended to exhibit stronger antioxidant activity as compared to purified fractions (GSP-1a), which was consistent with previous research that the free radical scavenging ability of pure polysaccharide from *Ziziphus jujuba* was lower than that of crude polysaccharide [33]. The difference in antioxidant activity could potentially be attributed to impurities present in the crude polysaccharide fraction, such as proteins, amino acids, organic acids, and other compounds that possess inherent antioxidant properties [57]. These impurities may contribute to the overall antioxidant capacity of the crude polysaccharide fraction.

### 2.6. Effect of GSP-1a on H_2_O_2_-Induced Oxidative Stress

#### 2.6.1. Effect of GSP-1a on the Change of ROS, MDA Content, and SOD Activities in H_2_O_2_-Treated HepG2 Cells

**Figure 6:**
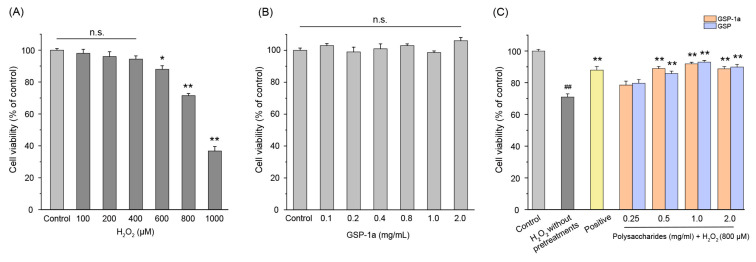
The effects of different concentrations of H_2_O_2_ on the viability of HepG2 cells (**A**), the effects of GSP-1a on HepG2 cells viability (**B**), and the protective effects of GSP-1a and GSP on HepG2 cells under H_2_O_2_-induced oxidative stress (**C**). The control group was normal cells without any treatment. The H_2_O_2_ without pretreatments group was only treated with 800 μM H_2_O_2_ for 4 h. The positive and sample groups were pretreated with Vc and polysaccharides for 24 h, respectively, and then treated with 800 μM H_2_O_2_ for 4 h. The data were expressed as the percentage of viable cells compared to the blank control and were presented as the mean ± SD (*n* ≥ 3). ^n.s.^ *p* > 0.05. ^##^ *p* < 0.01, in contrast to control group. * *p* < 0.05 and ** *p* < 0.01, in contrast to H_2_O_2_ without pretreatments group.

The hepatocellular carcinoma HepG2 cell has been widely used to evaluate antioxidant defense systems [33,58]. Thus, an oxidative stress model was established in HepG2 cells to assess the protective effect of GSP-1a. Initially, the optimal concentration of H_2_O_2_ required to induce oxidative stress in HepG2 cells was determined. As shown in Figure 6A, 600 μM or higher H_2_O_2_ concentrations decreased the percentage of living cells significantly compared with the control group. Considering that high H_2_O_2_ concentrations may cause cell death by non-apoptotic mechanisms [59], 800 μM H_2_O_2_ was selected to explore the protective effect of GSP-1a. According to Figure 6B, there was no significant difference in the viability of HepG2 cells when treated with a concentration range of 0 to 2 mg/mL of GSP-1a and GSP compared to the control group. This indicates that both GSP-1a and GSP concentrations were non-toxic to HepG2 cells. Consequently, different concentrations of GSP-1a (0.25, 0.5, 1, and 2 mg/mL) were selected to investigate their antioxidant activity in H_2_O_2_-induced oxidative stressed HepG2 cells. Based on the results shown in Figure 6C, the viability of HepG2 cells exhibited a significant increase when they were pretreated with GSP-1a and GSP, as compared to cells treated with H_2_O_2_ without any pretreatments. Notably, the viability of cells pretreated with 1 mg/mL GSP-1a reached 91.9% of the control value, and its protective effect was as good as the positive group. In addition, the cell viability under GSP-1a and GSP pretreatment were similar, and all were dose dependent. The results indicate that GSP-1a could be effective against H_2_O_2_-induced oxidative stress.

The addition of H_2_O_2_ can induce the production of excessive ROS in cells and excessive ROS further induces cell apoptosis [60]. ROS production serves as the most direct indicator for evaluating the antioxidative capacity. The lipid peroxide generated from the reaction between ROS and the double bond of polyunsaturated fatty acids forms a series of aldehyde compounds of which MDA is one of the main products, and excessive MDA can ultimately lead to disruption of cellular metabolism [61]. Therefore, the content of MDA can indirectly reflect the ROS-mediated cell oxidative stress degree. In addition, SOD converts superoxide anion free radicals into oxygen and hydrogen peroxide. The level of SOD reflects the intracellular antioxidant enzyme system load which serves as an indirect indicator for estimating the antioxidant effect; when oxidative stress is suffered, the activity of the SOD enzyme will decrease [62]. To elucidate the protective mechanism of GSP-1a on H_2_O_2_-mediated oxidative stress injury to HepG2 cells, ROS level and MDA content in HepG2 cells were investigated.

**Figure 7:**
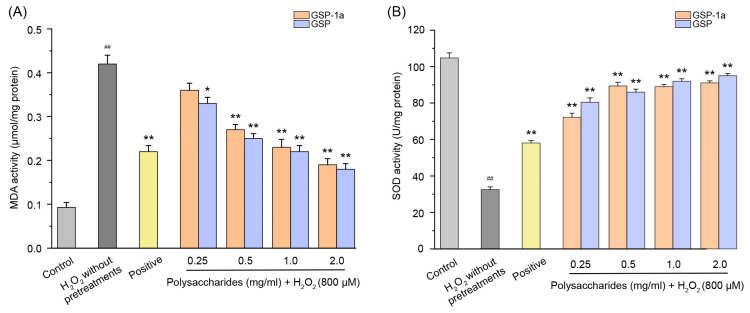
The MDA (**A**) and SOD (**B**) activities of HepG2 cells at different treatments. The control group was normal cells without any treatment. The H_2_O_2_ without pretreatments group was only treated with 800 μM H_2_O_2_ for 4 h. The positive and sample groups were pretreated with Vc and polysaccharides for 24 h, respectively, and then treated with 800 μM H_2_O_2_ for 4 h. The data were expressed as the means ± SD (*n* ≥ 3). ^##^ *p* < 0.01, in contrast to control group. * *p* < 0.05 and ** *p* < 0.01, in contrast to H_2_O_2_ without pretreatments group.

**Figure 8:**
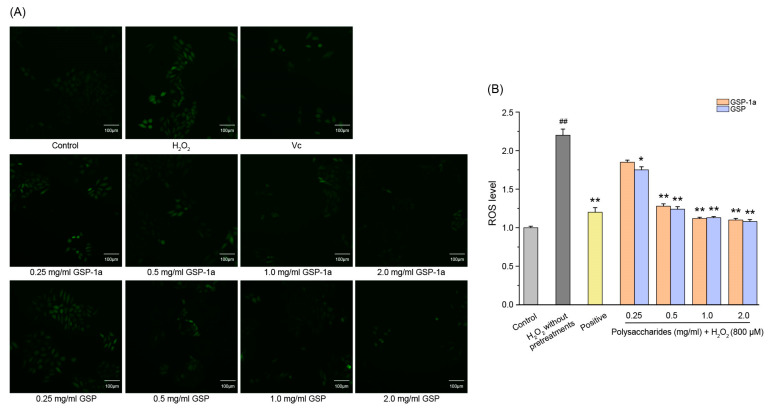
Effect of GSP-1a on ROS generation in HepG2 cells. DCF fluorescence intensity (**A**). Images of HepG2 cells captured by fluorescence microscopy (**B**). The control group was normal cells without any treatment. The H_2_O_2_ without pretreatments group was only treated with 800 μM H_2_O_2_ for 4 h. The positive and sample groups were pretreated with Vc and polysaccharides for 24 h, respectively, and then treated with 800 μM H_2_O_2_ for 4 h. The data were expressed as the means ± SD (*n* ≥ 3). ^##^ *p* < 0.01, in contrast to control group. * *p* < 0.05 and ** *p* < 0.01, in contrast to H_2_O_2_ without pretreatments group.

Intracellular ROS levels were determined using DCFH-DA, as shown in Figure 7A. The green fluorescence indicates that intracellular ROS formation was detected. In Figure 7B, the DCF fluorescence intensities of HepG2 cells treated with H_2_O_2_ without any pretreatments were significantly higher than those in the control group (*p* < 0.01), which indicated that H_2_O_2_ induced intracellular ROS generation. Compared to the H_2_O_2_ without pretreatments group, pretreatment with GSP-1a and GSP reduced DCF fluorescence intensity significantly. Notably, at a concentration of 0.5 mg/mL, GSP-1a exhibited a remarkable reduction in DCF fluorescence intensity, comparable to that of the positive group. In Figure 8A, a significant increase in MDA content was observed for cells treated with H_2_O_2_ without any pretreatment (0.42 μmol/mg) when compared to the control group, indicating the occurrence of oxidative stress and lipid peroxidation induced by H_2_O_2_. GSP-1a treatment led to significant alleviation of MDA levels in a dose-dependent manner. At a concentration of 2 mg/mL, the MDA content decreased to 0.19 μmol/mg, highlighting the effectiveness of GSP-1a in mitigating lipid peroxidation. Additionally, as depicted in Figure 8B, the SOD activity for cells treated with H_2_O_2_ without any pretreatment (32.5 U/mg) was significantly lower than that in the control group (104.8 U/mg) (*p* ˂ 0.01), suggesting that H_2_O_2_ caused damage to SOD. However, treatment with GSP-1a and GSP led to a significant increase in SOD activity compared to cells treated with H_2_O_2_ without pretreatments, showing a dose-dependent relationship. Notably, the SOD activity increased to 89.4 U/mg following pretreatment with 0.5 mg/mL GSP-1a. These results indicated that GSP-1a could protect against H_2_O_2_-induced oxidative stress in HepG2 cells by inhibiting intracellular ROS and MDA generation, as well as by improving SOD activity.

#### 2.6.2. Effect of GSP-1a on mRNA Expression of NRF2 and FNIP1 Pathway

**Figure 9:**
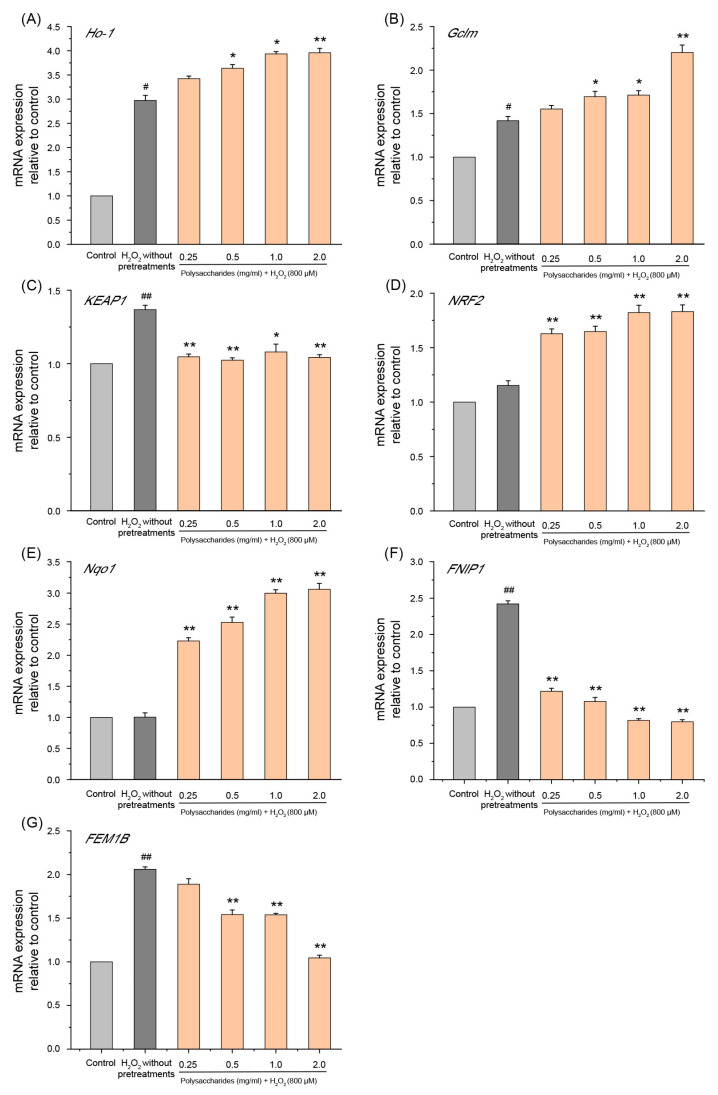
Effects of GSP-1a on *Ho-1* (**A**), *Gclm* (**B**), *KEAP1* (**C**), *NRF2* (**D**), *Nqo1* (**E**), *FNIP1* (**F**), and *FEM1B* (**G**) on mRNA expression. The control group was normal cells without any treatment. The H_2_O_2_ without pretreatments group was only treated with 800 μM H_2_O_2_ for 4 h. The sample group was pretreated with GSP-1a for 24 h, and then treated with 800 μM H_2_O_2_ for 4 h. The data were expressed as the means ± SD (*n* ≥ 3). ^#^ *p* < 0.05 and ^##^ *p* < 0.01, in contrast to control group. * *p* < 0.05 and ** *p* < 0.01, in contrast to H_2_O_2_ without pretreatments group.

Nuclear factor erythroid 2-related factor 2 (*NRF2*) is a critical transcription factor protecting against oxidative stress by regulating the transcription of several antioxidant genes, such as heme oxygenase-1 (*Ho-1*), quinone oxidoreductase 1 (*Nqo1*), and glutamate-cysteine ligase modifier subunit (*Gclm*) [63,64,65]. Kelch-like ECH-associated protein-1 (*KEAP1*) is an inhibitor of *NRF2* [66]. During oxidative stress, the *NRF2* moiety from *KEAP1* enters the nucleus to induce the expression of antioxidant genes [66]. In the current study, we found that the mRNA level of *Ho-1*, *Gclm*, and *KEAP1* was enhanced during treatment with H_2_O_2_ (Figure 9A–C). This is consistent with previous reports that antioxidant genes are expressed at low levels in cells under non-stimulated conditions but are rapidly induced by oxidants, and this enhanced expression plays a vital role in cellular protection under oxidative stress [67,68]. GSP-1a significantly increased the mRNA expression of *Ho-1*, *Gclm*, *Nqo1*, and *NRF2* in a concentration-dependent manner compared with cells treated with H_2_O_2_ without any pretreatment (Figure 9A–E). In addition, the pretreatment with GSP-1a reduced the mRNA expression of *KEAP1*, which contributes to alleviating the inhibitory effect of *KEAP1* on *NRF2*. These results demonstrate that GSP-1a may activate the *NRF2* pathway, upregulating downstream genes such as *Ho-1*, *Gclm*, and *Nqo1*, thereby protecting HepG2 cells from H_2_O_2_-induced oxidative stress. Additionally, Follicle-interacting protein 1 (*FNIP1*) and fem-1 homolog B (*FEM1B*) maintain mitochondrial redox homeostasis and are central components of the reductive stress response [69]. Recent studies have shown that the binding of FEM1B and FNIP1 proteins leads to the degradation of FNIP1, which activates ROS production in mitochondria in response to a reduction in ROS [70]. In this study, exposure to H_2_O_2_ led to a significant upregulation of mRNA levels for *FNIP1* and *FEM1B*, aligning with the non-physiological production of ROS. However, GSP-1a downregulated the expression levels of *FNIP1* and *FEM1B* in a concentration-dependent manner (Figure 9F,G), which may be due to the reduction in ROS production. Based on the above analysis, we propose that GSP-1a is likely to mitigate oxidative stress through the *NRF2* and *FNIP1* pathways.

## 3. Materials and Methods

### 3.1. Materials

*O. gracilis* fermentation spore powder was obtained from cultured *Paraisaria dubia* (anamorph of *O. gracilis*, CGMCC No. 20731, stored in China General Microbiological Culture Collection Center) according to the following methods. The strain was inoculated in the sporulation medium (glucose 20 g, yeast extract 30 g, KH_2_PO_4_ 2 g, MgSO_4_·7H_2_O 1 g, ZnSO_4_·7H_2_O 6 g, H_2_O 1 L, pH 8) and cultured for 12 days at 20 °C and 120 rpm. After fermentation, a small number of mycelia pellets were separated from the fermentation broth containing spores via filtration through a 200 mesh press cloth. Then, the fermentation broth was centrifuged at 8000 rpm for 10 min and the pellet was collected to obtain spore powder. The collected spore powder was lyophilized. The preparation of *O. gracilis* fermentation spore is shown in Figure S2.

DEAE Sepharose fast flow and Superdex G-200 were purchased from GE Healthcare Life Science (Piscataway, NJ, USA). Monosaccharide standards, 3-methyl-1-phenyl-2-pyrazolin-5-one (PMP), 1,1-diphenyl-2-picrylhydrazyl (DPPH^•^), 2,2′-azino-bis (3-ethylbenzothiazoline-6-sulphonic acid) (ABTS^•+^), and 3-(4,5-dimethylthiazol-2-yl)-2,5-diphenyltetrazolium bromide (MTT) were purchased from Sigma-Aldrich (Shanghai, China). Minimum essential medium (MEM), fetal bovine serum (FBS), and penicillin–streptomycin solution were obtained from KeyGEN Bio Tech (Nanjing, China). Alcohol, hydrogen peroxide (H_2_O_2_) solution, and dimethyl sulfoxide (DMSO) were obtained from Aladdin Industrial Corporation (Shanghai, China). ROS, MDA, SOD, and BCA protein concentration assay kits and cell lysis buffer for Western and IP were purchased from the Beyotime Institute of Biotechnology, Ltd. (Shanghai, China). HepG2 cells were purchased from Shanghai Cell Bank, Chinese Academy of Sciences (Shanghai, China).

### 3.2. Polysaccharides Extraction of O. gracilis Spores

#### 3.2.1. Preparation of Crude Polysaccharides

The spores were defatted with hexane and thereafter extracted with 0.25 M NaOH solution (1:15, *w*/*v*) at 90 °C for 3 h. The above alkaline extracts were neutralized with acetic acid and then centrifuged at 8000× *g* for 10 min to obtain the supernatant. Polysaccharides in the supernatant were precipitated using 4 times the volume of 95% ethanol at 4 °C for 12 h, followed by centrifugation at 8000× *g* for 10 min to separate the precipitated polysaccharides, and then washed using ethanol. In addition, the precipitate was deproteinized three times with ethanol to obtain crude polysaccharides (GSP). The deproteinization method was conducted as described before [71,72], with minor modifications, as follows. The precipitated polysaccharides were redissolved in distilled water then centrifuged at 8000× *g* for 10 min to remove precipitated denatured proteins. The supernatant was retained and precipitated polysaccharides using 4 times the volume of 95% ethanol at 4 °C; then, it was centrifuged to obtain polysaccharides precipitate (8000× *g*, 10 min). The above deproteinization process was repeated three times, and the last time, the polysaccharides precipitate was dissolved in water and then freeze-dried to obtain GSPs in the form of a powder. The yield of crude polysaccharides was calculated via the following:

(1) $$Yield\left(\%,w/w\right)=\frac{crude\;polysaccharides\;weight}{Raw\;material\;weight}\times 100.$$

#### 3.2.2. Isolation and Purification of Polysaccharides

The deproteinized polysaccharides were dissolved in distilled water and centrifuged (8000 rpm, 10 min); subsequently, they were elutied in DEAE Sepharose fast flow column (26 mm × 300 mm). GSP was gradient-eluted with 0, 0.1, and 0.3 mol/L NaCl at a flow rate of 1 mL/min. After 5 min, each tube was collected via an automatic fraction collector (BSZ-100, Huxi, China). Among the fractions collected, two peaks were detected by the phenol sulfuric acid method (GSP-1, GSP-2). The eluent of the two fractions was collected separately and was dialyzed to remove sodium chloride, free protein, and small-molecular-weight polysaccharides. Thereafter, the eluent of the two fractions was lyophilized using a vacuum dryer (Biosafer-10A, Biosafer, Nanjing, China) to obtain the polysaccharides in the form of a powder. GSP-1 was further purified with a Superdex G-200 gel column (16 mm × 600 mm) at a flow rate of 0.2 mL/min. One tube was collected every 20 min. The main fraction (GSP-1a) was collected and eluted samples were freeze-dried. The yield of pure polysaccharide was calculated via the following:

(2) $$Yield\left(\%,w/w\right)=\frac{Fractions\;weight}{Crude\;polysaccharides\;weight}\times 100.$$

### 3.3. Chemical Composition, UV Spectroscopy, and Molecular Weight Analysis

The phenol–sulfuric acid method was used to determine total sugar content with glucose as the standard. The protein content of GSP-1a was determined using a BCA protein concentration assay kit. Additionally, GSP-1a was dissolved in distilled water and subjected to UV–Visible spectroscopy in the range of 190–300 nm using a UV–Visible spectrometer (UV-T9, Persee, Beijing, China).

GSP-1a was dissolved in 0.1 M NaNO_3_ aqueous solution (0.02% NaN_3_, *w*/*w*) at a concentration of 1 mg/mL, and the solution was thereafter filtered through a 0.45 μm membrane. The molecular weight of GSP-1a was determined using high-performance gel permeation chromatography (HPGPC) with a binary HPLC pump (U3000, Thermo, Waltham, MA, USA) and a refractive index detector (Optilab T-rEX, Wyatt Technology, Co., St Milford, MA, USA). The polysaccharides solution was separated using three tandem columns (300 × 8 mm, Shodex OH-pak SB-805, 804, and 803; Showa Denko K.K., Tokyo, Japan) maintained at a temperature of 45 °C. The eluent employed for the separation was 0.1M NaNO_3_ containing 0.02% NaN3 (*w*/*w*). Each run involved injecting 100 μL of the sample at a flow rate of 0.5 mL/min.

### 3.4. Structural Characterization of GSP-1a

#### 3.4.1. FT–IR Analysis

The polysaccharide sample was prepared by grinding KBr to determine using FT–IR (Nicolet iS20, Thermo Fisher Scientific, Waltham, MA, USA) with a spectral range of 4000 to 400 cm^−1^.

#### 3.4.2. Monosaccharide Composition Analysis

The monosaccharide composition of GSP-1a was determined via high-performance anion-exchange chromatography (HPAEC). Briefly, 5 mg of the polysaccharide sample was hydrolyzed with 2 M trifluoroacetic acid (TFA) at 121 °C for 2 h in a sealed tube. The polysaccharide sample was dried with nitrogen then supplemented with methanol and blown dry to remove TFA. This process was repeated three times. The residue was re-dissolved in deionized water and filtered through 0.22 μm microporous filtering film for measurement. The processed sample was analyzed via high-performance anion-exchange chromatography (HPAEC) on a CarboPac PA-20 anion-exchange column (3 × 150 mm; Dionex, Sunnyvale, CA, USA) using a pulsed amperometric detector (Dionex ICS 5000 system, Thermo Fisher Scientific, Waltham, MA, USA) by Sanshu Biotech. Co., Ltd. (Shanghai, China). Flow rate, 0.5 mL/min; injection volume, 5 μL; solvent system A: (ddH_2_O), solvent system B: (0.1 M NaOH); solvent system C: (0.1 M NaOH, 0.2 M NaAc). The gradient program volume ratio of solution A, B, and C was 95:5:0 at 0 min, 85:5:10 at 26 min, 85:5:10 at 42 min, 60:0:40 at 42.1 min, 60:40:0 at 52 min, 95:5:0 at 52.1 min, and 95:5:0 at 60 min. Data were acquired on the ICS5000 (Thermo Fisher Scientific, Waltham, MA, USA) and processed using Chromeleon 7.2 CDS (Thermo Scientific, Waltham, MA, USA).

#### 3.4.3. Methylation Analysis

The GSP-1a was methylated according to the Ciucanu method with minor modifications [73]. GSP-1a was dissolved in anhydrous dimethyl sulfoxide (DMSO) and methylated in DMSO/NaOH with CH_3_I several times. After complete methylation, the permethylated products were hydrolyzed with 2 mol/L TFA at 121 °C for 1.5 h, reduced by sodium borodeuteride (NaBH_4_), and acetylated with acetic anhydride for 2.5 h (100 °C). After evaporating with toluene, the resulting methylated derivatives were analyzed with GC-MS on an Agilent 6890A-5975C equipped with Agilent BPX70 chromatographic column (30 m × 0.25 mm × 0.25 µm, SGE Analytical Science, Melbourne, Australia), and high purity helium (split ratio 10:1) was used as the carrier gas with an injection volume of 1 μL. Mass spectrometry analysis was performed at the initial temperature of 140 °C for 2 min, and the temperature was increased to 230 °C by 3 °C/min for 3 min. The scan mode was SCAN with a range (*m/z*) from 30 to 600.

#### 3.4.4. Nuclear Magnetic Resonance (NMR) Spectroscopy

The GSP-1a was dissolved in 0.5 mL D_2_O to a final concentration of 40 mg/mL. Recordings of 1D and 2D NMR (^1^H−NMR, ^13^C−NMR, ^1^H−^1^H COSY, ^1^H−^1^H NOESY, ^1^H−^13^C HMBC, and ^1^H−^13^C HSQC) were performed at 25 °C with a Bruker AVANCE NEO 500 M spectrometer system (Bruker, Rheinstetten, Germany), operating at 500 MHz, from Sanshu Biotech. Co., Ltd. (Shanghai, China).

### 3.5. Assay of Antioxidant Activity In Vitro

Hydroxyl radical (^•^OH), 2,2′-azino-bis-3-ethylbenzothiazoline-6-sulfonic acid radical cation (ABTS^•+^) and 2-diphenyl-1-picrylhydrazyl radical (DPPH^•^) scavenging ability of GSP-1a and GSP were measured to evaluate in vitro antioxidant activity. The ^•^OH, ABTS^•+^ and DPPH radical scavenging activity of polysaccharides were determined as described before [74,75,76]. Vc was used as the positive control.

### 3.6. Cellular Antioxidant Activity

#### 3.6.1. Cell Culture

Human hepatocellular carcinoma cell line HepG2 was cultured in Dulbecco’s Modified Eagle’s Medium (DMEM, Gibco, Waltham, MA, USA), supplemented with 10% FBS, 100 units/mL penicillin, and 100 units/mL streptomycin at 37 °C in a humidified atmosphere with 5% CO_2_.

#### 3.6.2. Oxidative Stress Model Induced by H_2_O_2_

In order to obtain stable experimental results, different concentrations (100, 200, 400, 600, 800, and 1000 μM) of H_2_O_2_ were treated in the HepG2 cells for 4 h to verify an appropriate concentration in the cell injury model. Cells without treatment were regarded as the control group. The cell viability was determined based on the MTT assay. We found that 800 μM H_2_O_2_ was sufficient to reduce the cell viability in HepG2 cells significantly.

#### 3.6.3. Treatment of Cells and Cell Viability Assay

HepG2 cells (1 × 10^4^ cells/mL, 100 μL) were cultured in 96-well plates for 24 h and then treated with GSP-1a (0, 0.1, 0.2, 0.4, 0.8, 1, and 2 mg/mL) for 24 h to evaluate its toxicity. The cell viability was determined based on the MTT assay.

After HepG2 cells were cultured for 24 h, the cells treated with different methods were divided into four groups: (1) the sample group consisted of cells pretreated with different doses of GSP-1a (0.25, 0.5, 1, 2 mg/mL) for 24 h, followed by treatment with 800 μmol/L H_2_O_2_ for 4 h; (2) the positive group included cells pretreated with 0.1 mg/mL Vc for 24 h, followed by treatment with 800 μmol/L H_2_O_2_ for 4 h; (3) the control group included the normal cells; (4) the H_2_O_2_ without pretreatments group consisted of cells which were only treated with 800 μM H_2_O_2_ for 4 h. After the different treatments above, the cell viability was evaluated via MTT assays, as described by Li et al. [77]. Each wall was supplemented with 10 μL MTT (0.5 mg/mL) for 4 h in an incubator with 5% CO_2_ at 37 °C. Then, the supernatant was removed, and the formazan was dissolved in DMSO. The absorbance at 570 nm was measured via a microplate reader. Cell viability was calculated using the following formula:

$$The\;cell\;viability\left(\%\right)=\frac{Ai}{Aj}\times 100$$

where A_i_ is the absorbance of the treatment group, and A_j_ is the absorbance of the blank control group (without treatment).

#### 3.6.4. Determination of ROS, MDA Content and SOD Activity

HepG2 cells were cultured in 6-well plates (1 × 10^6^ cells/mL, 2.5 mL) for 24 h then incubated with GSP-1a for 24 h followed by H_2_O_2_ (800 μM) for 6 h to determine ROS and MDA levels as well as the SOD activity.

The ROS level of the cells was determined using the hydrophilic probe 2′,7′-dichlorofluorescin diacetate (DCFH-DA). DCFH-DA permeates across the cell membrane and is de-esterified by cytosolic esterases to 2′,7′-dichlorodihydrofluorescein (DCFH), which is oxidized to 2′,7′-dichlorofluorescein (DCF) by ROS [36]. Oxidation is associated with an increase in green fluorescence. After completing the cell culture for the above four groups (control group, H_2_O_2_ without pretreatments group, sample group, positive group), they were washed in PBS buffer three times and then incubated with 1 mL of DCFH-DA probe (diluted by serum-free MEM culture medium with the final concentration of 10 μM) for 30 min (37 °C, 5%CO_2_). Subsequently, we removed the DCFH-DA solution, washed the cells three times with PBS buffer, and added 1 mL of PBS buffer to each well to keep the cells moist in preparation for measuring cell fluorescence intensity. Finally, the DCF fluorescence intensity was tested via a multi-detection microplate reader (Synergy, Biotek Instruments, Inc., Winooski, VT, USA) with an excitation wavelength of 488 nm and an emission wavelength of 525 nm. All these experiments were also performed in triplicate. The final statistical results were expressed as a percentage of the control group. In addition, HepG2 cells in the four groups were observed via inverted fluorescence microscope (Olympus-IX73P2F, Tokyo, Japan).

The MDA content was measured using a kit according to the manufacturer’s protocol. Cells were washed three times with PBS buffer and lysed with Western and IP cell lysis buffer. The supernatant was then collected via centrifuging at 4 °C, 12,000× *g* for 5 min. MDA was determined using the thiobarbituric acid-reactive substances (MDA-TBA adduct). Samples (0.1mL) were treated with TBA reagent (6.32 mM TBA, 0.2mL) and then heated at 100 °C for 15 min. Subsequently, we centrifuged the reaction mixture, took 200 µL of the supernatant into a 96-well plate, and measured the absorbance at 532nm. The results are expressed in terms of protein content per unit weight (µmol/mg protein).

The intracellular SOD activity was determined using a total SOD assay kit with WST-8, according to the manufacturer’s instructions. Cells were washed three times with PBS buffer and lysed with Western and IP cell lysis buffer. The supernatant was then collected via centrifuging at 4 °C, 12,000× *g*, for 5 min. We mixed the SOD detection buffer, WST-8 solution, and enzyme solution in proportion to prepare the WST-8/enzyme working solution and added 160 μL to each well of a 96-well plate. The sample group was supplemented with 20 μL sample solution and 20 μL reaction initiation solution, blank control hole 1 was supplemented with 20 μL SOD detection buffer and 20 μL reaction initiation solution, the blank control hole 2 was supplemented with 40 μL SOD detection buffer, while the blank control hole 3 was supplemented with 20 μL SOD detection buffer and 20 μL samples. Next, the solution was incubated at 37 °C for 30 min, and absorbance at 450 nm was measured. We calculated enzyme activity (U/mg protein) based on the protein content of the sample.

The concentration of protein was also measured via a BCA protein assay kit according to the manufacturer’s protocols. The absorbance was determined by the microplate reader (Biotek Synergy, Winooski, VT, USA). All these experiments were also performed in triplicate.

#### 3.6.5. Total RNA Extraction and qRT-PCR

The total RNA of the control, H_2_O_2_ model, and sample group cells were extracted via Trizol Kit according to the manufacturer’s instructions. Total RNA was reverse transcribed into cDNA with HiScript II Q RT SuperMix. Real-time quantitative polymerase chain reaction (qPCR) was performed on QuantStudio 3 (Thermo Fisher Scientific, Waltham, MA, USA) by using SYBR Green master mix, with each sample prepared in triplicate. The above kits and reagents were purchased from Vazyme Biotech Co., Ltd., Nanjing, China. The primer sequences were shown in Table S1, and *GAPDH* was used as a reference gene. The relative expression level of the mRNAs was calculated using the 2^−ΔΔCT^ method.

### 3.7. Statistical Analysis

The data from three independent experiments are presented as means ± SD. The GraphPad Prism 8.0 (GraphPad Software, Inc., San Diego, CA, USA) was used to conduct one-way ANOVA tests for inter-group comparison. *p* < 0.05 and *p* < 0.01 were considered statistically significant.

## 4. Conclusions

In this study, we isolated a neutral heteropolysaccharide, GSP-1a (72.8 kDa), from *O. gracilis* spores, which mainly consisted of mannose, galactose, and glucose. GSP-1a was composed of α-D-Galp-(1→, →6)-α-D-Manp-(1→, →2,6)-α-D-Manp-(1→, →6)-α-D-Glcp-(1→, →3,6)-α-D-Glcp-(1→ and →2,4,6)-α-D-Manp-(1→ residues, and its partial hypothetical model structure was inferred. Furthermore, GSP-1a exhibits protective effects against H_2_O_2_-induced oxidative stress in HepG2 cells. This protective mechanism involved the regulation of the NRF2 and FNIP1 pathways. These findings highlight the antioxidant activity of polysaccharides derived from *O. gracilis* fermentation spores.

## Figures and Tables

**Table 1:** Molecular weights and chemical compositions of GSP-1a.

Samples	GSP-1a
Yield/%	33.5 ± 0.64
Total carbohydrate (%)	82 ± 1.5
Protein (%)	ND
Nucleic acid (%)	ND
Mw (kDa)	72.8
Mw/Mn	1.1
Monosaccharide composition (molar ratio)	
Man	42.28%
Gal	35.7%
Glc	22.02%

“ND” refers to “not detected”.

**Table 2:** Methylation analysis data for GSP-1a.

Retention Time (min)	Type of Linkages	PMAAs	Relative Molar Ratio (%)	Mass Fragments (*m/z*)
8.477	t-Gal(p)	1,5-di-O-acetyl-2,3,4,6-tetra-O-methyl galactitol	34.36	60, 71, 87, 102, 118, 145, 162, 205
11.847	2-Man(p)	1,2,5-tri-O-acetyl-3,4,6-tri-O-methyl mannitol	1.95	60, 71, 88, 118, 129, 145, 161, 190
13.074	6-Man(p)	1,5,6-tri-O-acetyl-2,3,4-tri-O-methyl mannitol	19.99	71, 87, 99, 102, 118, 129, 162, 173.1, 189
13.153	6-Glc(p)	1,5,6-tri-O-acetyl-2,3,4-tri-O-methyl glucitol	9.87	60, 87, 102, 118, 129, 137, 162, 173, 189
13.508	4-Glc(p)	1,4,5-tri-O-acetyl-2,3,6-tri-O-methyl glucitol	3.68	60, 87, 102, 118, 129, 137, 162, 191
17.316	3,6-Glc(p)	1,3,5,6-tetra-O-acetyl-2,4-di-O-methyl glucitol	9.60	71, 87, 101, 118, 129, 137, 49, 174, 189, 202, 234, 245, 299
17.610	2,6-Man(p)	1,2,5,6-tetra-O-acetyl-3,4-di-O-methyl mannitol	15.46	71, 87, 99, 118, 129, 149, 167, 189, 202, 218, 261, 299, 338
18.292	2,4,6-Man(p)	1,2,4,5,6-penta-O-acetyl-3-O-methyl mannitol	5.09	60, 74, 88, 117, 130, 159, 190, 202, 218, 233.1, 260, 299, 338

**Table 3:** The detailed ^1^H and ^13^C NMR spectral assignments of GSP-1a.

Code	Glycosyl Residues		Chemical Shifts (ppm)					
			1	2	3	4	5	6
A	α-D-Gal*p*-(1→	H	4.92	3.50	3.74	3.66	4.01	3.71, 3.8
		C	97.29	71.24	72.09	72.68	74.37	60.39
B	→6)-α-D-Man*p*-(1→	H	5.08	3.95	4.05	3.75	3.78	3.77
		C	106.28	74.37	74.72	72.34	80.94	70.65
C	→2,6)-α-D-Man*p*-(1→	H	5.27	4.19	4.11	3.79	3.78	3.75, 4.28
		C	98.97	79.72	76.88	70.65	80.94	72.36
D	→6)-α-D-Glc*p*-(1→	H	4.84	3.50	3.71	3.94	4.17	3.39, 3.83
		C	99.56	73.06	72.81	76.75	75.31	69.46
E	→3,6)-α-D-Glc*p*-(1→	H	4.98	3.51	4.16	3.94	4.17	3.91, 3.56
		C	97.24	71.03	86.89	76.75	75.31	69.59
F	→2,4,6)-α-D-Man*p*-(1→	H	5.27	4.19	4.05	3.96	ND	3.76, 4.28
		C	99.09	79.72	74.72	82.23	ND	72.09

“ND” refers to “no signals were detected”.

## Data Availability

The data presented in this study are available in the article.

## Supplementary Materials

The following supporting information can be downloaded at: https://www.mdpi.com/article/10.3390/ijms241914721/s1.
