# Supplementary material for: A Neutral Polysaccharide from Spores of Ophiocordyceps gracilis Regulates Oxidative Stress via NRF2/FNIP1 Pathway
Source: Int J Mol Sci. 2023 Sep 29;24(19):14721. doi: 10.3390/ijms241914721 (PMC10572349; doi:10.3390/ijms241914721)
Supplement: Supplementary file 1 [file ijms-24-14721-s001.zip › ijms-2575118-supplementary.pdf]

Supplementary Material

A neutral polysaccharide from spores of *Ophiocordyceps gracilis* regulates oxidative stress via NRF2/FNIP1 pathway

Yue Wang, Shixiang Wei, Hui Lian, Lingling Tong, Linhui Yang, Bo Ren, Dongsheng Guo * and He Huang *

School of Food Science and Pharmaceutical Engineering, Nanjing Normal University, NO 1, Wenyuan Road, Nanjing 210023, China.

*** Correspondence:**
Dongsheng Guo
guodongs@njnu.edu.cn

He Huang
huangh@njnu.edu.cn

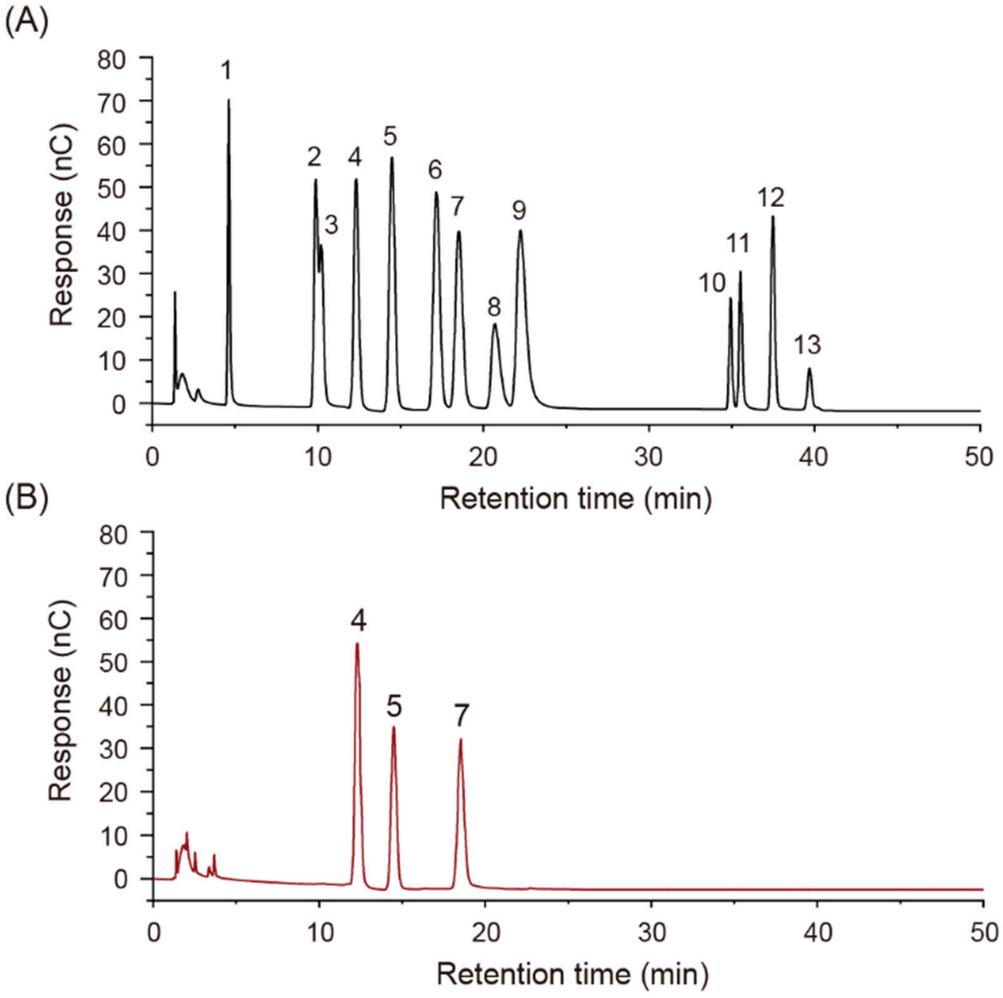

Figure S1. The HPAEC chromatograms of standard monosaccharides (A) and GSP-1a monosaccharide components (B). (1. Fuc, 2. Ara, 3. Rha, 4. Gal, 5. Glc, 6. Xyl, 7. Man, 8. Fru, 9. Rib, 10. Gal-UA, 11. Gul-UA, 12. Glc-UA, 13. Man-UA).

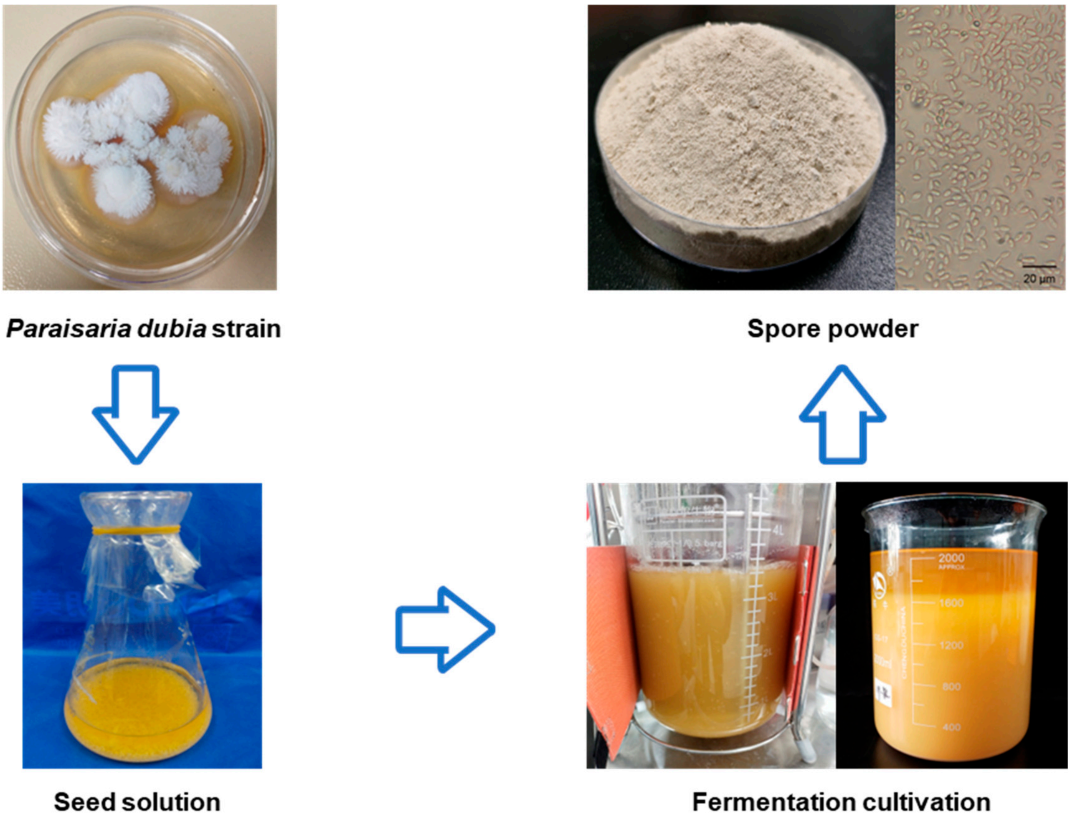

Figure S2. The preparation of *Ophiocordyceps gracilis* fermentation spore powder.

Table S1 Primer sequences

	Forward primer	Reverse primer
Ho-1	GCCAGCAACAAAGTGCAAGA	TAAGGACCCATCGGAGAAGC
Gclm	GACAAAACACAGTTGGAACAGC	CAGTCAAATCTGGTGGCATC
Nqo1	TGGTGGAGTCGGACCTCTATG	CATGGCAGCGTAAGTGTAAGC
NRF2	GCCTGTAAGTCCTGGTCATCG	GCTCTTTGGACATCATTTCGTTG
KEAP1	TGTCCTCAATCGTCTCCTTTATGC	CCGCTTCGGATGGTGTTCATT
FNIP1	TGAAAGTGAAGATACAGGTCATGATA	CAGCACTCACTTCGATTAACTTTG
FEM1B	CACTCCATCATCATTAGCCTAGTTGA	TGTACTTTTGTCTAGCGGAGTCTTATTCT
GAPDH	CGACCACTTTGTCAAGCTCA	AGGGGTCTACATGGCAACTG
